# On Practical Nosology

**Published:** 1819-03

**Authors:** 


THE LONDON

### Medical and Physical Journal.

3 OF VOL. XLI.] MARCH, 1819. [NO. 241.

"For many fortunate discoveries in medicine, and for the detection of numerous errors, the world is indebted to the rapid circulation of Monthly Journals; and there never existed any work to which the Faculty in EUROPE and AMERICA were under deeper obligations than to the Medical and Physical Journal of London, now forming a long, but an invaluable, series."—RUSH.

*For the London Medical and Physical Journal.*

### *On Practical Nosology*; by Dr. KINGLAKE.

THE nominal designation of disease has undeniably many scientific, as well as practical, advantages; yet it must be confessed that the difficulty of distinguishing correctly, and of avoiding injurious conclusions, is so great as to render it extremely doubtful whether the prevailing nosology of the day is not more hurtful than beneficial. Every experienced practitioner must be fully aware of the incalculable importance of accurately discriminating between one disease and another. Were all morbid affections radically similar, however they might vary in external appearances, it would be of less moment to come to a true decision on the nature of an occurring disease, than it evidently is under the essential differences actually existing in the several disorders incident to human nature.

Every scheme of nosology that has been proposed seems to have been built on the unstable ground of considering every variety of constitution to be sufficiently similar to admit of general distinctions, as to the nature and character of occurring diseases. This persuasion is erroneous, and has served to mislead the opinions and judgments of men, whose caution and discernment were entitled to much respect. Gaubius, Sauvages, Vogel, Cullen, &c. have severally presented nosological arrangements, that have been adopted, and have become, in a greater or less degree, popular, as their respective advocates have found reason to be satisfied with their practical correctness and utility. Weighty objections have been felt and urged against the accuracy of the distinctions proposed by those nosologists; but that which is most authoritative, is the impossibility of grouping any set of symptoms that can uniformly and conclusively distinguish one disease from another. Unhappily for the interest of the medical art, diseases have been arranged under certain denominations that are supposed to convey precise distinctions as to the particular nature and tendency of the disease so named. It is obvious that no name can fully describe the real character of a disease; and, if it could, the circumstances necessary to produce such a disease will not present with sufficient constancy to warrant the application of such a name. If the name, therefore, possesses no certainty on which could be infallibly rested a real distinction of disease, the instances of misnomer in nosological designation must be so frequent as greatly to lessen, if not wholly to destroy, their practical utility.

Nosologists do not appear to have duly considered the changeful state of vital action in the progress of disease, but to have gratuitously imagined that the same condition of living power prevails during the whole course of a morbid process. It is notorious that ailments commence with inflammatory violence, and speedily run into a state of debility that at once reverses the nature and character of the disease. Were the treatment that would be proper for inflammatory violence to be continued under the fallacious guide of a name implying that state, the result would probably be destructive of life. In low remittent fever,—in dyspeptic irritation, with its manifold and extensive sympathies,—in the various painful affections proceeding from the debilitated, tremulous, and irregular action called spasm,—much of the inflammatory aspect of disease may arise; and, if the symptoms should coincide with the factitious grouping that nosologists have held to be characteristic of that state, a mode of treatment would be instituted that would be more likely to aggravate than to benefit the complaint. The vascular struggles connected with a morbidly-irritable state, proceeding from great debility, are often so active and so acutely painful as to induce a suspicion of their being inflammatory. Were such symptoms to be so denominated, it would suggest a mode of treatment that could not fail to be seriously detrimental.

In disordered sensibility, from the highest state of mania to the lowest ebb of feeling, various degrees of morbid sensation will arise, in which ample range is afforded for distinguishing what instances may be referrable to deficient, and what to excessive, nervous energy. It would require an acumen surpassing the utmost nosological skill to apportion the precise force, and peculiar form of disease, that could be intelligibly expressed by any name that could be employed. The different degrees of affection presenting under such circumstances must be sought out, and ascertained by direct and close observation; from whence alone can be inferred conclusions that would be safely and usefully practical on the subject.

The intelligent physiologist must not regard medical nomenclature as a landmark for his direction, nor as a nominal epitome of the various forms of diseased action. He must take his stand on higher ground, and be decided only by actual occurrences, deliberately examined by the most guarded and attentive observation. From this source alone can be derived an adequate authority for appreciating the nature, and determining the treatment, of disease.

The usage of name, in considering and reporting disease, is so familiar, that it must be complied with to a certain extent. Popular explanations will require it; but it is an object devoutly to be wished that medical practitioners would not be influenced by mere name, but be deliberately occupied in investigating the true nature and character of the disease, which alone can suggest such indications of cure as would be either worthy of science to adopt, or safe for the patient to undergo. No one conversant with the practice of medicine can doubt the numerous instances that occur of the disastrous influence of name in the treatment of disease. Too much attention is commonly directed to the class, the order, or variety of affection, to which an ailment should be referred; an anxious desire is shown to determine by what name the complaint should be distinguished. When this point has been settled, the treatment is confidently founded on the admitted correctness of the adopted name. Thus, if the disease is held to be inflammatory, and if one viscus is more particularly affected than another, the affection is named accordingly: if the brain, it is called phrenitis; the heart, carditis; the lungs, pneumonia; the stomach, gastritis; the intestines, enteritis; the liver, hepatitis, &c. &c. Should the notion entertained of the state of the disease be correct, the nominal designation given to the particular viscus that may have either originated or concentrated the force of the disease might be practically useful; but, if any fallacy should arise in regarding the disease as inflammatory, and if it should prove to be radically spasmodic and of typhoid tendency, the treatment that would be applicable to the one would be inadmissible in the other; yet it is known how far the authority of a mere name often goes in governing the management of disease. To preclude the risk that must necessarily attend medical practice founded on the nominal distinction of disease, it would be better to enter on the treatment under the strict guidance of the symptoms that may actually occur, leaving the epithet of name out of consideration, until the progress of the affection shall have developed appearances too minutely to admit of any possible doubt as to the true and explicit designation of the ailment.

The name of a disease, abstractedly considered, is no more than that which is attached to any other object, for the convenience of particular distinction: it conveys no satisfactory information of the nature of a disease, any more than the name of an animal, a vegetable, or a mineral, does the intrinsic and characteristic qualities of those several substances. The assemblage of symptoms that is supposed to be expressed by a nosological appellation may not be individually and collectively present; and, if they were, they furnish no certain criterion by which to estimate the true nature and character of the disease. The difficulty is for ever recurring respecting the vast variety of constitutional action that might have induced the prevailing symptoms. If constitutions, widely varying in degrees of vital power and active resources, should, under certain circumstances of diseased excitement, produce symptoms very analogous to each other, yet the fundamental dissimilarity of their origin must be accompanied by a corresponding difference in their respective nature and mode of cure. Symptoms evincing undiminished vital power immoderately excited, however classed or named, too obviously require suitable reduction to need any nominal distinction to enforce it; and, where an enfeebled state of vital power is inordinately excited, the indication of cure will be afforded by considering the extent to which the vital energy may have been diminished, independently of any claims arising from any name that may have been given to the attending symptoms.

In fact, and in truth, diseases themselves, and not their artificial designation, are the legitimate objects of medical practice; and, although nosological arrangements may be creditable to the benevolence and industry of science, it will not be denied that they tend, generally speaking, rather to confound and mislead than to elucidate difficulties, and clearly point out an appropriate mode of treatment in the various and equivocal aspects of morbid affection to which human existence is incessantly liable.

*Taunton; Sept.* 19*th*, 1818.

